# Mechanisms and clinical progress of adipose-derived stem cells and their derivatives in the treatment of hair loss

**DOI:** 10.1186/s13287-025-04560-7

**Published:** 2025-08-08

**Authors:** Jiale Zhang, Feng Chen, Yue Hu, Xianling Cong

**Affiliations:** 1 Department of Dermatology, China-Japan Union Hospital of Jilin University, Changchun, 130033 China; 2 Department of Biobank, China-Japan Union Hospital of Jilin University, Changchun, 130033 China

**Keywords:** Alopecia, Hair regeneration, Stem cell therapy, Adipose-derived stem cells, Exosomes

## Abstract

The rising prevalence of alopecia poses a significant challenge for both clinicians and researchers. As the global incidence of hair loss continues to increase, research into hair biology and regenerative mechanisms has gained considerable attention. However, current treatment options for alopecia are often constrained by limited efficacy and notable adverse effects. This underscores an urgent need for innovative therapeutic strategies to address these gaps. Adipose-derived stem cells (ADSCs), a subset of mesenchymal stem cells, represent a promising new approach in the treatment of alopecia. This review provides a detailed examination of the fundamental properties of ADSCs and their derivatives, exploring their mechanisms of action in alopecia therapy. Analysis of the efficacy of ADSCs and their derivatives in both preclinical and clinical settings highlight their potential to stimulate hair regeneration. Additionally, the review discusses various pre-treatment methods designed to enhance the regenerative capacity of ADSCs in hair growth, elucidating the mechanisms involved. The review also addresses the challenges and future directions for the use of ADSCs in alopecia treatment, aiming to offer valuable insights for both theoretical research and clinical practice. Ultimately, this work seeks to contribute to the development of more effective treatment regimens for alopecia.

## Introduction

Hair loss is a common condition caused by a variety of factors, including endocrine disorders, stress, nutritional and metabolic imbalances, diseases and infections, genetic predispositions, and aging [1]. Studies suggest that over 50% of the global population will experience some degree of hair loss in their lifetime, with androgenetic alopecia (AGA) and alopecia areata (AA) being the most prevalent types. Notably, AGA accounts for 90% of all hair loss cases [2]. The underlying mechanism of AGA involves hair follicles being highly sensitive to dihydrotestosterone (DHT), an androgen metabolite, which leads to the gradual shrinkage of hair follicles, shortening of the anagen phase, and eventually, hair loss [2]. This condition not only affects physical appearance but also significantly impacts the mental health of affected individuals, often leading to social isolation, depression, and anxiety [3–5].

Currently, the FDA has approved only two drugs for the treatment of AGA: finasteride and minoxidil [6]. Although these drugs are effective in slowing hair loss, they come with potential side effects and can lead to dependence with long-term use [7]. In addition to these medications, other commonly employed treatment modalities include dutasteride, platelet-rich plasma (PRP), hair transplantation, low-dose oral minoxidil (LDOM), low-level laser therapy (LLLT), botulinum toxin type A (BTX-A), and micro needling. However, these approaches exhibit significant limitations in terms of efficacy, safety, and applicability [8–14]. Compounding these challenges, approximately 20 million patients do not respond to traditional treatments, creating an urgent need for new therapeutic options [15].

Stem cell therapy has emerged in recent years as a promising approach to treating a variety of diseases [16]. Stem cells are characterized by their ability to self-renew and differentiate into multiple functional cell types under specific conditions, allowing them to repair and replace damaged tissues. Because of these properties, stem cells have broad applications in treating various conditions, including hematologic disorders, burns, bone defects, and cardiovascular diseases [17–19]. For example, stem cells can promote lung tissue repair by differentiating into various lung cell types [20]. Moreover, stem cells can be used to treat conditions such as pneumonia, sepsis, and acute lung injury by inhibiting inflammatory responses, reducing vascular leakage, and enhancing bacterial clearance [21–24]. In the context of immunomodulation, stem cells secrete immunosuppressive cytokines like IL-7, IL-11, IL-14, and IL-15, which have been used in the treatment of autoimmune diseases such as systemic lupus erythematosus, rheumatoid arthritis, and multiple sclerosis [25, 26].

In recent years, stem cell-based therapies have demonstrated considerable promise in the treatment of hair loss. Transplantation of various stem cell types—including those derived from adipose tissue, dental pulp, hair follicles, and umbilical cord—has been shown to promote hair regeneration [27–29]. Among these, ADSCs have gained considerable attention in the field of alopecia treatment due to their wide availability, ease of access, ability to be auto transplanted, pluripotency, and safety profile [30]. Multiple studies have demonstrated that ADSCs possess significant potential and efficacy in treating hair loss conditions, particularly alopecia areata. In summary, this review will focus on exploring the mechanisms and clinical progress of adipose-derived stem cells and their derivatives in the treatment of hair loss (Tables 1 and 2). The aim is to provide insights into their clinical applications and to highlight potential future research directions and opportunities for therapeutic development.

**Table 1:** ADSCs and derivatives in hair regeneration

Component	Affected cells/tissues	Mechanism	Effects	Up/ Downward	Ref.
ADSCs	Follicular cells	PDGF	The anagen phase of the hair follicle cycle	↑	[39]
	Follicular cells	HGF	Follicular pigmentation and proliferation	↑	[40]
	Follicular cells	VEGF	Neovascularization	↑	[41]
	DPCs	IGF-1	Follicular cell proliferation	↑	[42, 43]
	-	IL-6	Number of hair follicles	↑	[45]
	Follicular cells	FGF-1 、FGF-6	Number of hair follicles	↑	[44]
	NK cells, T cells, B cells	Inhibition of NK, T, and B cells	Folliculitis	↓	[51]
	Dermal fibroblasts, Epidermal keratinocytes	Antioxidant proteins and enzymes↑	Antioxidation	↑	[53, 54]
	Follicular cells	Androgen antagonist	DPC	↑	[22]
ADSC-CM	Follicular cells	Growth factors	Hair	↑	[57]
	Follicular cells	HFDPCs、HEKs	Hair	↑	[57]
	Follicular cells	Wnt signaling, growth factors	Transition into the growth phase	↑	[58]
	Follicular cells	Antioxidant proteins and enzymes↑	Folliculitis	↓	[59]
ADSC-Exos	DPCs	-	Number of DPCs	↑	[63]
	-	ALP、VCAN、β-catenin、LEF-1	hair follicle regeneration	↑	[64]
	Follicular cells	Activation of the Erk and Akt Signaling pathways	Follicular cell proliferation	↑	[65]
	Follicular cells	Upregulation of Cyclin D1	Regulation of hair follicle cell cycle		[66, 67]
	DPCs, follicular cells	miR-122-5p	DHT suppression of DPC and hair Follicles	↓	[68]
	Hair follicles affected by AGA	miR-122-5p, Inhibition of the TGF-β/SMAD3 pathway.	Regeneration of hair follicles in AGA	↑	[68]
	Follicular cells	The expression of β-catenin and proteoglycans. ↑	Normal follicular growth	↑	[68]

**Table 2:** Clinical advances of ADSCs and their derivatives

Component	Sample	Treatment method	Effects	Up/downward	Ref.
ADSCs	AGA patient	adipose tissue + ADRCs	number of hairs in the low-dose group	↑	[75]
	AGA patient	adipose-derived stem cell extract	hair density and thickness	↑	[76]
ADSC-CM	AGA patient	ADSC-CM + fractional laser/microneedling	hair regeneration rate	↑	[77]
	AGA patient	ADSC-CM + non-ablative fractional laser	hair density and count	↑	[78]
	AGA, FPHL	Intradermal injection of freeze-dried powder of ADSC-CM secreted proteins	hair density and count	↑	[80]
	AGA patient	ADSC-CM + antioxidant/finasteride injection	hair density and thickness	↑	[81]
	patient with alopecia	intradermal injection of ADSC-CM	hair density and count	↑	[82]
SVF	AA patient	ADSVCs	hair density and diameter	↑	[83]
	FPHL, MPHL	Fat + SVF injection	hair count	↑	[84]
	AGA patient	SVF + PRP injection	hair density and count	↑	[85]
	AGA patient	SVF + PRP	hair density and keratin score	↑	[86]
	AGA patient	Autologous SVF + Finasteride/Dutasteride/Minoxidil	hair density and keratin score	↑	[73]
ADSC-Exos	patient with alopecia	ADSC-Exos microneedle injection	hair density and thickness	↑	[75]
	mouse experiments	ADSC-Exos injection	Promoting follicle maturation and hair regeneration, increased expression of PDGF and VEGF, and decreased expression of TGF-β1	↑↓	[62, 88]

## Adipose stem cells and hair regeneration

### Regulation of the hair follicle cycle by ADSCs

Hair growth follows a cyclical process known as the hair follicle cycle, which consists of the anagen (growth), catagen (regression), and telogen (resting) phases. Altering this cycle can effectively promote hair growth, for example by prolonging the anagen phase, delaying catagen, or encouraging the transition from telogen to anagen [31]. ADSCs have demonstrated substantial potential in promoting hair growth, making them a promising option for treating hair loss [32]. ADSCs have been shown to stimulate the proliferation and migration of hair follicle cells, extend the anagen phase, and induce the differentiation of follicular stem cells [32, 33]. Research has revealed that several cytokines secreted by ADSCs play a critical role in promoting hair regeneration. These include vascular endothelial growth factor (VEGF), insulin-like growth factor (IGF), hepatocyte growth factor (HGF), platelet-derived growth factor (PDGF), bone morphogenetic protein (BMP), interleukin-6 (IL-6), macrophage colony-stimulating factor (M-CSF), endothelial cell growth factor (ECGF), fibroblast growth factor − 1 (FGF-1), fibroblast growth factor-6 (FGF-6), alkaline phosphatase (ALP) and others [34–38]. For instance, PDGF has been shown to induce and maintain the anagen phase in mouse models [39]. HGF may enhance hair follicle pigmentation and proliferation by increasing β-catenin expression through paracrine signaling [40]. VEGF accelerates hair follicle development and regrowth by promoting vascularization around the follicles [41], while IGF-1 directly influences dermal papilla cells (DPCs), restoring their ability to induce hair growth and improving follicle cell migration, survival, and proliferation [42, 43]. FGF-1 and FGF-6 can stimulate hair follicle proliferation and contribute to the anagen phase of the hair cycle [44]. A study reported that high levels of IL-6 secreted by ADSCs increase cell proliferation and the number of hair follicles, further promoting hair regeneration [45]. Moreover, DKK1-knockout ADSCs (DKK1-KO-ASCs) have been shown to activate the Wnt signaling pathway and secrete increased levels of growth-promoting factors, thereby significantly enhancing the proliferation of hair follicle-associated cells, such as outer root sheath (ORS) cells, and ultimately improving hair regeneration [46]. In summary, ADSCs effectively promote hair regrowth through multiple mechanisms and pathways, highlighting their potential as a treatment for hair loss.

### Anti-inflammatory effects of ADSCs

ADSCs have demonstrated significant anti-inflammatory effects in various immune-related diseases, such as Crohn’ s disease, osteoarthritis, and acute respiratory distress syndrome [47–49]. These anti-inflammatory properties are primarily attributed to the secretion of extracellular vehicles (EVs), which play a key role in modulating the immune response. This mechanism also shows great potential for hair regeneration. Many forms of hair loss, including androgenetic alopecia, are often accompanied by localized scalp inflammation, fibrosis, and immune cell infiltration around the hair follicles [50]. ADSCs can effectively reduce inflammation and fibrosis, improving the follicular microenvironment and promoting hair regeneration. Zhao et al. found that ADSCs inhibit the proliferation and differentiation of natural killer (NK) cells and T cells, reducing their cytotoxic effects. At the same time, they suppress B cell maturation and antibody production, weakening the inflammatory response and protecting hair follicles from damage [51]. These findings not only deepen our understanding of the role of ADSCs in immunomodulation but also provide new perspectives and strategies for disease treatment and regenerative medicine.

### Antioxidant effects of ADSCs

ADSCs exhibit significant antioxidant properties, which further contribute to their effectiveness in promoting hair growth. They secrete various antioxidant proteins, such as pigment epithelium-derived growth factor (PEDF), hepatocyte growth factor (HGF), interleukin-1(IL-1), granulocyte-macrophage colony-stimulating factor (GM-CSF), peroxidase, and insulin-like growth factor-binding protein (IGFBP) [52]. These proteins provide antioxidant and anti-apoptotic protection to dermal fibroblasts and epidermal keratinocytes, shielding them from free radical damage. Moreover, ADSCs help eliminate intracellular reactive oxygen species (ROS) by accelerating mitochondrial autophagy and enhancing the expression of antioxidant enzymes like catalase and superoxide dismutase (SOD). This leads to improved mitochondrial function, increased cellular resistance to oxidative stress, and reduced cellular damage [53, 54].

The therapeutic potential of ADSCs in hair loss management is substantiated by their multifaceted antioxidant strategy. Experimental evidence reveals their ability to counteract androgen-induced damage by reducing ROS-mediated injury to hair follicle epithelial cells, suppressing apoptosis, and stimulating dermal papilla cell proliferation [22]. In conclusion, ADSCs effectively alleviate local oxidative stress in hair follicles, protect them from free radical-induced damage, and reduce hair follicle degeneration. Their antioxidant properties make ADSCs a powerful tool for mitigating hair loss through various mechanisms, including the secretion of antioxidant proteins, enhancement of mitochondrial autophagy, and increased expression of antioxidant enzymes.

### Neovascularized effects of ADSCs

The blood supply to the scalp determines the nutritional status of hair follicles, which in turn influences the progression of conditions like alopecia areata. In cases of androgenetic or scarring alopecia, improving the local blood supply to the scalp can promote hair regeneration. Growth factors secreted by ADSCs play a crucial role in neovascularization by influencing the activity of surrounding cells [55]. Xiong BJ et al. showed that after transplanting 0.3 ml of ADSC suspension along with 1 ml of adipose tissue into mice, the concentration of vascular endothelial growth factor (VEGF) increased, which was accompanied by a rise in blood vessel density within the adipose tissue [56]. This suggests that ADSCs can enhance the local blood supply around hair follicles by secreting factors like VEGF, thereby promoting hair regeneration. These findings indicate that ADSCs can support hair regeneration by improving the local blood supply around hair follicles.

## Adipose stem cell derivatives and hair regeneration

### Conditioned media for ADSCs

The culture fluid obtained during the isolation, extraction, and expansion of ADSCs is called ADSC-conditioned media (ADSC-CM). This medium contains various growth factors, such as Insulin-like growth factor binding protein-1 (IGFBP-1), Insulin-like growth factor binding protein-2 (IGFBP-2), macrophage colony-stimulating factor (M-CSF), M-CSF receptor, platelet-derived growth factor receptor-β (PDGF R-β), vascular endothelial growth factor (VEGF), and epidermal growth factor (EGF). Furthermore, studies have demonstrated that ADSC-CM can promote the proliferation of human hair follicle dermal papilla cells (HFDPCs) as well as human epidermal keratinocytes (HEKs) [57]. Additionally, ADSC-CM promotes the transition of hair follicles from the resting phase to the anagen phase through the Wnt signaling pathway [58]. It also attenuates damage caused by inflammation and aging in hair follicles and surrounding tissues due to its antioxidant components, such as glutathione peroxidase (GPx), superoxide dismutase (SOD), and thrombopoietin (TPO) [59]. Overall, ADSC-CM demonstrates great potential in hair loss treatment by regulating the cell cycle, activating key signaling pathways, providing antioxidant protection, and enhancing cell migration and tissue repair. Moreover, using conditioned media eliminates ethical concerns associated with cell culture and handling, as well as the risk of immune rejection or tumor formation that may arise from cell transplantation.

### Stromal vascular fraction of ADSCs

The stromal vascular fraction (SVF) is a heterogeneous mixture of cells extracted from adipose tissue. It includes pericytes, endothelial cells, macrophages, and other immune cells [60]. One significant advantage of SVF is its ability to isolate sufficient cell populations in real time, without requiring complex culture expansion. This makes SVF an ideal material for cell-based therapies [48]. SVF contains not only growth factors but also ADSCs, which have been shown to promote hair growth significantly [55]. In addition to its role in neovascularization, SVF reduces inflammatory responses. The anti-inflammatory properties reduce the inflammation surrounding hair follicles, while the anti-androgenic effects counteract the adverse influence of androgens on hair follicles, thus promoting healthy hair growth [60, 61]. In summary, SVF is a mixture rich in growth factors and stem cells that not only promotes hair growth but also offers therapeutic benefits through its anti-inflammatory and angiogenesis.

### Adipose-derived stem cell exosomes (ADSC-Exos)

Adipose-derived stem cell exosomes (ADSC-Exos) play an active role in the treatment of hair loss [34, 62]. ADSC-Exos are considered one of the most effective methods for increasing hair induction rates in DPCs both in vivo and in vitro [63]. A study showed that ADSC-Exos increased the proliferation and survival of DPCs while maintaining their hair-inductive capacity [34]. Research demonstrates that ADSC-Exos upregulate the expression of hair growth-related genes, such as alkaline phosphatase (ALP), versican (VCAN), β-catenin, and LEF-1. Concurrently, they activate the Wnt/β-catenin signaling pathway, enhancing β-catenin accumulation to promote hair follicle development and hair regeneration [64] Furthermore, ADSC-Exos promote the proliferation of hair follicle cells by activating the Erk and Akt signaling pathways [65]. They also regulate the cell cycle of hair follicle cells by upregulating the expression of cell cycle protein D1 and reduce apoptosis induced by H_2_O_2_ [66, 67]. Recent research has shown that miR-122-5p carried by ADSC-Exos counteracts the inhibitory effects of DHT on DPCs and hair follicles, stimulating the proliferation and migration of DPCs. However, studies on the role of miR-122-5p remain limited, and its underlying mechanisms require further validation through experimental and clinical investigations Additionally, ADSC-Exos significantly promote the regeneration of androgenetic alopecia (AGA) hair follicles by inhibiting the TGF-β/SMAD3 pathway [68]. Compared to traditional ADSC and ADSC-CM treatments, exosomes offer potential advantages, including being cell-free and less likely to provoke immune rejection [69]. Previous studies have shown that DPC-derived exosomes can induce the transition of hair follicles from the resting phase to the anagen phase in mice, while also prolonging the anagen phase [70]. These effects are mediated by the β-catenin signaling pathway [71]. Exosomes enriched with Wnt3a, Wnt11, Wnt4, and β-catenin proteins may enhance hair growth by promoting Wnt/β-catenin signaling [72]. Furthermore, hydrophobic Wnt proteins carried on the surface of exosomes can induce the activation of β-catenin, a key signaling pathway in hair morphogenesis and regeneration [73]. ADSC-Exos have also been shown to restore hair bulb size and increase dermal thickness by upregulating the expression of β-catenin and multifunctional proteoglycans, which promotes normal hair follicle growth [68]. Moreover, ADSC-Exos not only support healthy hair growth but also mitigate the inhibitory effects of DHT on hair growth, presenting new possibilities for AGA treatment [74]. These findings suggest that exosomes not only directly promote hair growth by delivering specific proteins but also enhance hair follicle regeneration by activating key signaling pathways. Thus, ADSC-Exos offer promising new strategies for future hair loss treatment.

## Adipose-derived stem cell therapy for hair regrowth

### Adipose-derived stem cells

ADSCs show great potential in promoting hair regrowth. A prospective trial involving 71 androgenetic alopecia (AGA) patients revealed nuanced therapeutic outcomes across treatment groups. While the low-dose ADSC group (0.5 × 10⁶ ADRCs/cm² + fat grafting) achieved superior hair counts at 24 weeks, the high-dose cohort (1.0 × 10⁶ ADRCs/cm²) showed diminished efficacy, potentially due to excessive macrophage recruitment triggering counterproductive microinflammation [75]. Further studies confirm that ADSC-derived components enhance both hair thickness and density, particularly benefiting early-stage AGA progression [76].

Unlike conventional treatments, ADSC therapies combine biological precision with minimized adverse effects. The observed dose-dependent outcomes emphasize optimized delivery strategies to maximize therapeutic ratios while avoiding inflammatory cascades. Clinical data collectively position ADSCs as a dual-action solution—promoting follicular regeneration through cellular activation while maintaining superior safety profiles compared to pharmacological or surgical alternatives. This evidence-based paradigm shift supports ADSC integration as either a primary or adjuvant therapy, offering patients a clinically validated, minimally invasive option for sustainable hair restoration.

### Adipose-derived stem cell derivatives

#### Conditioned medium for ADSCs

ADSC-conditioned medium (ADSC-CM) is considered to have great potential for promoting hair regeneration due to its rich content of growth factors and cytokines. Studies have shown that ADSC-CM combined with carbon dioxide fractional laser or microneedling can effectively treat alopecia areata (AA) [77]. The topical application of ADSC-CM combined with a non-stripping fractional laser significantly accelerated the increase in hair density and follicle number in AGA patients [78]. Its synergistic potential is further evidenced by co-administration with minoxidil, which amplifies hair parameter improvements within six weeks, suggesting cross-pathway potentiation [79]. Freeze-dried ADSC-CM formulations from healthy donors, delivered via intradermal injection, enhance hair counts in both AGA and female pattern hair loss (FPHL), while combinatorial protocols with finasteride achieve comparable efficacy without compromising safety [80, 81]. These multimodal approaches leverage ADSC-CM’s growth factor-cytokine axis to optimize follicular microenvironment remodeling.

Standardized monotherapy regimens using microneedling or mesotherapy devices over 12 weeks yield consistent hair density gains in male and female pattern alopecia, validating its standalone therapeutic capacity [50]. Intracranial ADSC-CM injections in AA patients not only boost hair density but also rebalance hair cycle dynamics, increasing anagen-phase ratios—a critical metric for sustained regrowth [82]. Crucially, ADSC-CM circumvents systemic side effects associated with pharmacological agents, as evidenced by zero adverse events across trials involving antioxidant-enriched or drug-combined formulations [81]. This safety-efficacy equilibrium, coupled with adaptable administration routes (topical, injectable, or device-assisted), positions ADSC-CM as a precision-tailored solution for diverse alopecia subtypes, bridging regenerative biology with clinical pragmatism.

#### Vascular stromal components of ADSCs

Stromal vascular fraction (SVF) has emerged as a potent regenerative tool for hair loss, with clinical studies underscoring its ability to enhance hair density, diameter, and cycle dynamics. Autologous adipose-derived stromal vascular cell (ADSVC) therapy significantly boosted hair regeneration in alopecia areata (AA), with marked improvements in density and shaft thickness at 3–6 months, particularly among female patients [83]. Comparative trials reveal SVF’s superiority over fat grafting alone, with SVF-enriched fat injections driving superior anagen-phase progression and follicular reactivation [84]. Synergistic protocols, such as SVF combined with platelet-rich plasma (PRP) to form platelet-rich substrates (PRS), have restored functionality to dormant follicles, inducing new hair growth in androgenetic alopecia (AGA) patients after a single treatment [85]. Further evidence from a six-month study showed SVF-PRP combinations elevating hair density and keratin scores, while retrospective analyses of SVF monotherapy (alongside oral/topical agents) reported 48% density improvements on treated scalp regions [60, 86]. These outcomes highlight SVF’s versatility across delivery methods and patient demographics.

Despite promising results, SVF therapy faces translational hurdles. Limited long-term safety data and heterogeneous patient responses necessitate larger, population-specific clinical trials to validate durability and tolerability. Additionally, the high cost of SVF isolation and processing raises concerns about accessibility, urging research into streamlined protocols or adjuvant strategies to enhance cost-effectiveness. Current studies, while demonstrating SVF’s compatibility with pharmacological agents like finasteride and minoxidil, also underscore the need to define optimal dosing regimens and combination therapies to maximize therapeutic synergy. Addressing these gaps will be pivotal for integrating SVF into mainstream alopecia management while ensuring equitable patient access.

#### Exosomes for ADSCs

Exosomes have become widely used in hair loss treatment due to their cell-free nature and lack of immune rejection. In a study, microneedling of ADSC exosomes into the scalp of 39 patients over 12 weeks resulted in significant improvements in hair density and thickness [87]. The study further confirmed that ADSC exosomes significantly promote hair follicle maturation and hair regeneration in mice, while increasing the expression of PDGF and VEGF and decreasing the expression of transforming growth factor-β1 (TGF-β1) [50]. The decrease in TGF-β1 expression may help maintain hair growth, as this factor is involved in follicular regression and apoptosis-related pathways [88, 89]. These results indicate that ADSC exosomes hold significant potential for effectively promoting hair regrowth.

#### Stimulating the hair regeneration potential of ADSCs

**Fig. 1:**
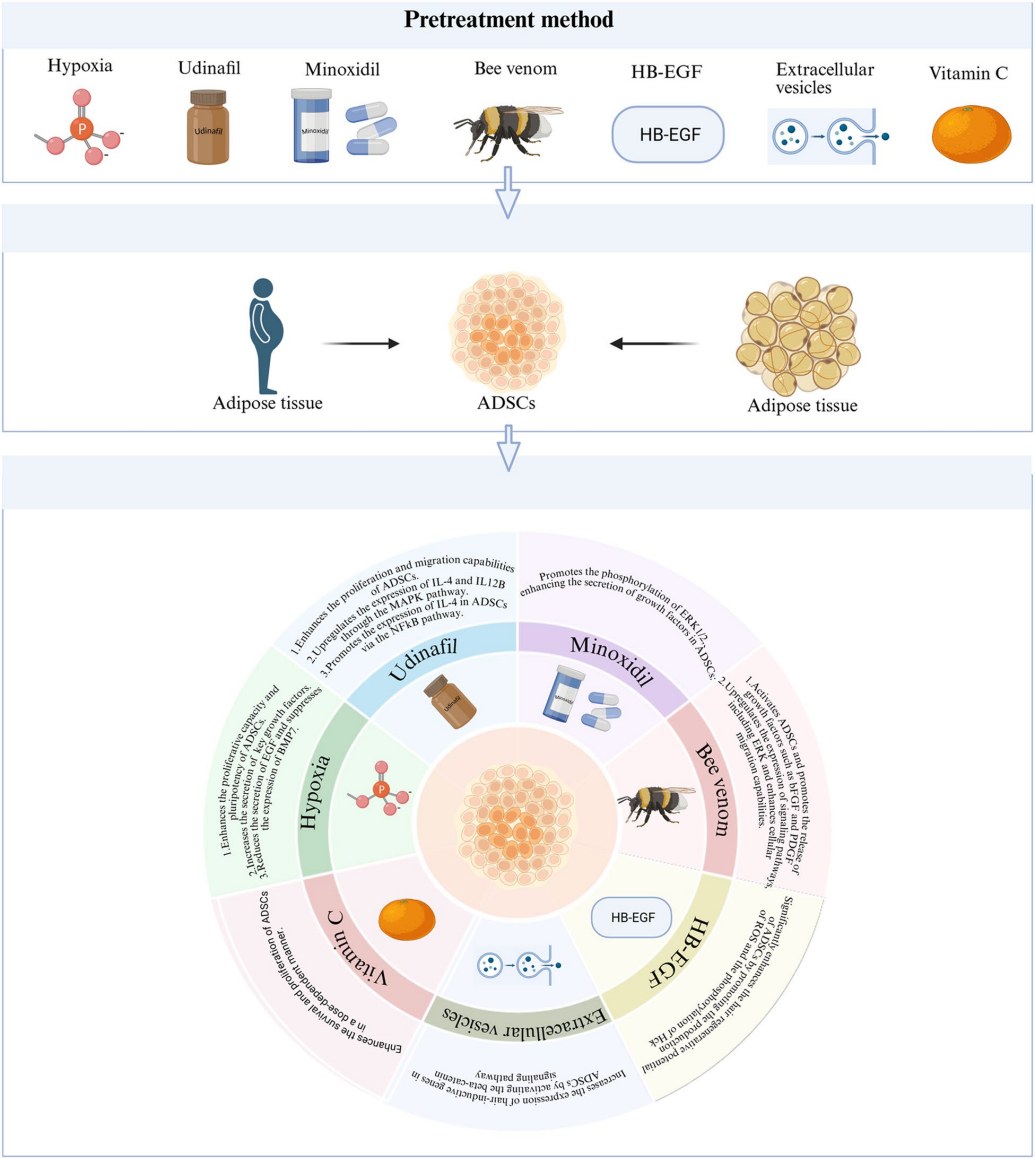
Pretreatment enhances the hair regeneration potential of ADSCs. Pretreatment methods including hypoxia, udenafil, minoxidil, bee venom, HB-EGF, extracellular vesicles, and vitamin C optimize the regenerative effects of ADSCs through multiple mechanisms

Not only can ADSCs and their derivatives treat hair loss, but pre-treatment—such as with hypoxia, udenafil, or minoxidil—can significantly enhance their hair regeneration capacity, offering new avenues to improve their therapeutic efficacy [90–93] (Fig. 1). Hypoxic preconditioning reshapes ADSC paracrine activity by upregulating pro-regenerative factors (bFGF, PDGF, VEGF) while suppressing inhibitory signals (EGF, BMP7), thereby enhancing folliculogenic potential [90, 91]. Pharmacological priming further optimizes ADSC functionality: Udenafil drives IL-4/IL-12B overexpression via MAPK/NFκB pathways, boosting dermal papilla cell (DPC) recruitment and activity [92], while minoxidil activates ERK1/2 to elevate PD-ECGF and PDGF-C secretion, improving follicular angiogenesis and proliferation [93]. Natural adjuvants like bee venom synergize with ADSCs by upregulating ERK-mediated bFGF/PDGF release and cell migration, increasing hair weight in murine models [94]. Complementary approaches—including HB-EGF-induced Hck phosphorylation for ROS-mediated activation [95], β-catenin-activated extracellular vesicles for hair-inductive gene expression [96], and vitamin C’s dose-dependent enhancement of ADSC viability—collectively establish a toolkit for precision-enhanced regenerative therapy [97].

These multimodal strategies demonstrate that ADSC efficacy is not static but dynamically programmable through microenvironmental or biochemical cues. By selectively amplifying growth factor cascades (CXCL1 for cell homing, IGFBP-1/2 for follicular maintenance) and neutralizing inhibitory pathways (TGF-β/SMAD3, BMP7), preconditioned ADSCs achieve superior spatial and temporal control over hair cycle regulation. However, clinical implementation requires balancing synergistic combinations—e.g., hypoxia-minoxidil co-treatment for angiogenic and proliferative synergy—with scalable manufacturing protocols. Future research must address dose optimization, delivery vehicle design (e.g., extracellular vesicle encapsulation), and long-term safety profiles to transform these mechanistically robust approaches into standardized, cost-effective therapies. Such advances will position preconditioned ADSCs as a next-generation platform for personalized alopecia management.

## Conclusion and outlook

**Fig. 2:**
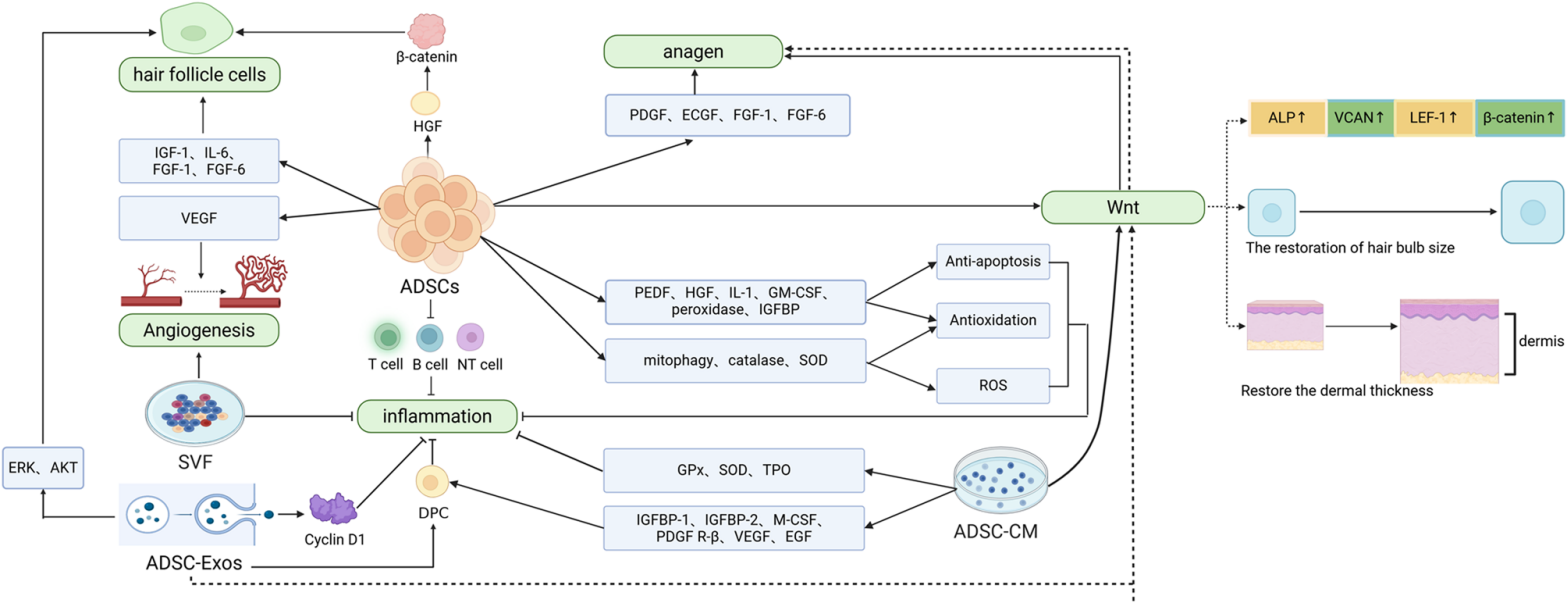
The mechanism of ADSCs and derivatives in hair regeneration. It highlights essential processes, including the modulation of the inflammatory microenvironment, activation of hair follicle stem cell proliferation, the initiation of signaling pathways, secretion of growth factors, and tissue regeneration

Because of various mechanisms, ADSCs and their derivatives have shown remarkable potential for application in hair regeneration (Fig. 2). ADSCs promote hair growth and follicular regeneration through various mechanisms, including regulation of the hair follicle cycle, anti-inflammatory actions, antioxidant effects, and neovascularization [32, 51–53]. In addition to ADSCs, their derivatives also play important roles in hair regeneration. ADSC-CM promotes hair regrowth by stimulating cell proliferation, activating the Wnt signaling pathway, and alleviating follicular damage [58, 59, 91]. Moreover, the SVF facilitates hair follicle repair and regeneration by secreting a variety of growth factors, exerting anti-inflammatory effects, and promoting angiogenesis [55, 60, 61]. Notably, ADSC-Exos can not only directly stimulate hair growth by delivering specific proteins but also enhance follicular regeneration through the activation of key signaling pathways [39, 65–68].

Currently, several treatments are available for hair loss, each presenting distinct advantages and limitations (Table 3). In the field of stem cell research, umbilical cord-derived mesenchymal stem cells (UC-MSCs) have attracted much attention due to their low immunogenicity, stable source, and broad regenerative potential [137]. Studies have shown that UC-MSCs promote tissue repair and hair regrowth, especially in patients with alopecia areata, with visible hair regrowth within 1 to 3 months post-treatment [138]. UC-MSC-derived exosomes (UCMSC-Exos) also exhibit similar regenerative effects, possibly by activating follicular stem/progenitor cells and the Wnt/β-catenin signaling pathway [139]. However, clinical application remains limited by the lack of long-term efficacy data, unclear therapeutic mechanisms, and technical challenges in cell expansion [138, 139]. Dental pulp stem cells (DPSCs) are another promising candidate due to their easy accessibility, rapid proliferation, and low immunogenicity [143]. SHED-conditioned medium (SHED-CM) has shown significant therapeutic effects in androgenetic alopecia (AGA), independent of dihydrotestosterone (DHT) inhibition [142,]. SHED-CM promotes anagen-phase follicle proliferation and reduces the proportion of follicles in telogen, with faster onset of action compared to follicle stem cell-conditioned media [141]. Under hypoxic conditions, DPSC-conditioned medium (H-CM) has shown potential in accelerating regrowth and improving hair quality in chemotherapy-related alopecia models [27]. However, most studies remain preclinical, and challenges such as limited clinical evidence, cell harvesting constraints, and long-term efficacy assessment continue to hinder widespread clinical application [27, 141, 143, 148].

**Table 3:** Advances in the treatment strategies for hair loss

Drug	Mechanism	Administration Route	Cost	experimental period	Efficacy	Advantages	Side Effects	Ref.
Minoxidil	Vasodilation、Anagen phase of the hair follicle↑	Topical	Low	24weeks	Hair density ↑	Applied by oneself	Contact dermatitis, hirsutism, poor compliance	[98–101]
Finasteride	5-alpha reductase inhibitors	Oral	Low	6 months	Hair quantity ↑	Convenient, postmenopausal FPHL	Sexual dysfunction, psychological disorders, risk of prostate cancer↑	[102–105]
Dutasteride	5-alpha reductase inhibitors	Oral	Low	6 months	Hair quantity↑	Oral finasteride non-responders	Nasopharyngitis, erectile dysfunction, decreased libido	[9, 106–108]
PRP	Growth Factor↑	Subcutaneous, intradermal, microneedle injection	High	12 weeks	Hair quantity, density ↑, negative rate of hair pull test is 91.7%	Hair pull test and satisfaction are superior to the minoxidil group	Erythema, edema, transient ecchymosis, infection、pigmentation, cervical lymphadenopathy, serum sickness, skin nodular lesions, irreversible monocular blindness, scalp sensitivity	[10, 109–116]
Hair Transplantation	Hair Follicle Count↑	FUT, FUE, ARTAS	High	-	Hair density self-esteem and satisfaction ↑	FUG: Less invasive, fast healing, inconspicuous scarring	Keloid, crusting, frontal edema, aseptic folliculitis	[11, 117–119]
LDOM	Vasodilation	Oral	Low	3–6 months	Hair density and quantity ↑	High convenience and compliance	Orthostatic hypotension, fluid retention, tachycardia, pericarditis and nausea, hirsutism, pedal edema	[12, 120–123]
LLLT	anti-inflammatory	Helmet-type, Laser comb	Medium	3–6 months	Hair density and thickness ↑	Mild adverse reactions, non-invasive	Transient alopecia, pruritus, tenderness and acne	[124–128]
BTX-A	DHT-induced TGF-β1 secretion↓	Intradermal injection, Intramuscular injection, Subcutaneous injection	Medium	3 months	The effective rate is 70%-79%, hair quantity ↑	Minoxidil allergic individuals, more effective on the forehead and temples	Headache, pain and erythema at the injection site	[13, 129–132]
Microneedling	Wnt/β-catenin↑, absorption rate↑, tissue repair↑	Microneedling	Medium	6 months	Hair quantity, density, hair shaft diameter ↑	Transdermal absorption ↑	Pain, transient punctate bleeding, erythema, lateral cervical lymphadenopathy	[14, 112, 133–136]
UC-MSCs	DPLTs, Hair Follicle Regeneration↑	Intradermal injection	High	1–3 months	1–3 months: AA cured, 45 days: Hair follicles ↑	No invasive surgery required	Insufficient clinical research	[137–140]
SHED-CM	Anagen hair follicles↑	Intradermal injection	High	31–59 days	75% effective	Easy acquisition, low immunogenicity	Minor bleeding, pinprick pain	[27, 141–143]
HFSCs	Hair follicle repair	Interfollicular infiltration Injection, Multi-point injection	High	1 months, 58 weeks	Hair diameter, proportion, count, and density↑	Hair count and density ↑	Some effects are temporary, redness and swelling at the injection site	[144–146]
ADSCs	Regulate the cell cycle, anti-inflammation, antioxidation, promote angiogenesis	Dermal and subcutaneous injection, Topical	High	6–16 weeks	Hair quantity and diameter ↑	Wide source, easy to obtain, autologous transplantation feasible, pluripotency	The mechanism is unclear, long-term efficacy and safety are unknown	[30, 75, 76, 147]

Finally, while ADSCs have made considerable advancements, several challenges remain. Firstly, the molecular mechanisms by which ADSCs and their derivatives regulate hair regrowth are not fully understood, especially regarding the validation of their long-term effects and safety [146]. Secondly, there is a need to optimize the standardized extraction methods, administration routes, and dosing regimens for ADSCs and their derivatives to enhance therapeutic efficacy while minimizing risks and ensuring the safety and consistency of clinical applications [149]. Future research should prioritize the following areas: optimizing ADSC extraction and application techniques, exploring the specific mechanisms of ADSCs in various types of hair loss, and conducting large-scale, randomized controlled trials to validate their efficacy and safety, with clearly defined indications and contraindications [150]. Furthermore, advancements in gene editing and nanotechnology hold promise for developing more stable and efficient ADSC-based therapies [151]. The combination of ADSCs with other therapeutic approaches, such as laser treatments, drugs, and microneedling, is expected to further enhance clinical outcomes [152]. In conclusion, ADSCs and their derivatives hold great promise as novel therapeutic tools in the treatment of hair loss, particularly alopecia areata. However, additional basic research and clinical validation are needed to establish their role as a safe and effective treatment. With ongoing advancements in science and technology, ADSCs have the potential to revolutionize the treatment of alopecia and offer more effective treatment options for patients.

## Data Availability

The data that support the findings of this study are available from the corresponding author upon reasonable request.

## Declarations

### Generative AI and AI-assisted technologies in the writing process

The authors declare that they have not use AI-generated work in this manuscript.
